# Pigment Integrity-to-Dust Ratio (PIDR): A Novel Bioindicator for Assessing Urban Air Pollution Stress in *Ginkgo biloba*

**DOI:** 10.3390/plants15121893

**Published:** 2026-06-18

**Authors:** Semonti Mukherjee, Dina Bibi, Bianka Sipos, Vanda Éva Abriha-Molnár, László Orlóci, Szilvia Kisvarga, Katalin Horotán, Zsanett Istvánfi, Viktor Oláh, Béla Tóthmérész, Tibor Magura, Edina Simon

**Affiliations:** 1 Department of Ecology, University of Debrecen, H-4032 Debrecen, Hungary; semonti-mukherjee@mailbox.unideb.hu (S.M.); bibi.dina@science.unideb.hu (D.B.); sipos.bianka@science.unideb.hu (B.S.); molnarvandaeva@science.unideb.hu (V.É.A.-M.); magura.tibor@science.unideb.hu (T.M.); 2 HUN-REN–UD Anthropocene Ecology Research Group, University of Debrecen, H-4032 Debrecen, Hungary; 3 Ornamental Plant and Green System Management Research Group, Institute of Landscape Architecture, Urban Planning and Garden Art, Hungarian University of Agriculture and Life Sciences, H-1223 Budapest, Hungary; orloci.laszlo@uni-mate.hu (L.O.); kisvarga.szilvia@uni-mate.hu (S.K.); horotan.katalin@uni-eszterhazy.hu (K.H.); istvanfi.zsanett@uni-mate.hu (Z.I.); 4 Institute of Biology, Eszterházy Károly Catholic University, 3300 Eger, Hungary; 5 Doctoral School of Plant Sciences, Hungarian University of Agriculture and Life Sciences (MATE), 1223 Budapest, Hungary; 6 Department of Botany, University of Debrecen, H-4032 Debrecen, Hungary; olahviktor@unideb.hu; 7 HUN-REN-UD Functional and Restoration Ecology Research Group, University of Debrecen, H-4032 Debrecen, Hungary; tothmeresz.bela@science.unideb.hu

**Keywords:** *Ginkgo biloba*, photosynthetic pigments, chlorophyll, carotenoids, particulate matter, urban pollution, plant stress

## Abstract

This study focused on the spatial and temporal changes in photosynthetic pigment concentrations in the leaves of *Ginkgo biloba* and their integration into a new bioindicator index, the Pigment Integrity-to-Dust Ratio (PIDR), to assess urban air pollution stress on trees in Budapest, Hungary. High levels of chlorophyll and carotenoids in early summer indicated greater pigment integrity at the moderate-traffic site, whereas there were clear indications of reductions in the high-traffic area. The control site represented a low-traffic, pollution-free baseline. Chlorophyll concentrations dropped in the traffic-exposed leaves, and there were increased levels in the formation of pheophytin. It is thought that these reductions were caused by city stress. Responses of pigments were also variable at the moderate site, perhaps due to some form of recovery or adjustment in the study’s time frame. The observed negative relationships between selected pollutants and PIDR suggested that pollutant exposure was associated with pigment degradation and foliar dust deposition, although these associations should be interpreted as exploratory. The Air Pollution Tolerance Index (APTI) was significantly different between the pollution-exposed sites and the control, reflecting physiological tolerance in chronically exposed trees rather than directly measuring pigment damage. Therefore, the APTI and PIDR provide complementary information. Overall, the PIDR appears to be a promising exploratory bioindicator of physiological stress response, based on pigment concentration changes and dust deposition.

## 1. Introduction

Air pollution poses a serious threat to urban and natural environments and human health [1,2]. More than 99% of the world’s population is exposed to PM_2.5_ levels exceeding World Health Organization recommendations [2]. The urban system is heavily influenced by pollution, driven by the increasing use of transport vehicles and industrial emissions [3,4]. Airborne particles, such as PM2.5, and atmospheric pollutants, including NO_2_, SO_2_, O_3_, and CO, negatively affect plant physiology by slowing photosynthesis, altering pigment composition, decreasing stomatal performance, and delaying foliage development, ultimately leading to leaf senescence and shortening the plant’s life [5,6,7,8,9,10,11]. In cities, air pollution leads to heat islands, fluctuation in surface moisture, and higher nighttime temperatures. The European Economic Area (2023) reported that, although SO_2_ emissions declined after the late 20th century, permitted levels of NO_2_, O_3_, and PM_2.5_ are still exceeded in the vicinity of traffic corridors [12,13]. Particulate matter (PM_10_, PM_2.5_) and gaseous pollutants (NO_2_, SO_2_, O_3_, CO) often accumulate along traffic corridors because vehicular emissions are the chief emission source in the city. Particulate matter is generally accumulated on the external surface of leaves, while gaseous pollutants enter leaf tissues via stomatal openings. PM affects plant health by covering stomatal pores, thereby altering stomatal functioning and reducing solar irradiance, which, in turn, affects the absorption of incident solar light, while gaseous pollutants directly affect chlorophyll content and accelerate pheophytin accumulation. PM also supplies trace metals that may lead to the generation of reactive oxygen species in plant leaves [5].

A higher proportion of internal combustion automobiles and less stringent pollution requirements for residential heating put central and eastern European cities, such as Budapest, in a vulnerable position. These cities are also exposed to stronger urban heat islands than areas that benefit from oceanic airflows, as pollutants can become trapped in the boundary layer due to topography [14]. Along with topography, pollutants vary seasonally; summer increases in O_3_ levels are related to photochemistry, while particulate matter levels are higher during the peak cold season due to winter heating [15,16].

Fixed stations located according to the European Union Ambient Air Quality Directive, with predetermined monitoring regulations, cannot detect subtle changes in pollutant levels [17,18]. While they do provide data for high-level analysis, they cannot interpret it in relation to effects on the biological system. This has led to increased appreciation for biomonitoring as a complementary process, with these stations providing a more comprehensive understanding [5,19,20]. Plants are considered effective bioreporters for various monitoring approaches, but city trees are vital for increasing the air-cleaning capacity of urban areas as they not only protect the microclimate but also absorb substantial amounts of gaseous air emissions [21,22,23]. Photosynthetic pigments, which contribute to many chemical processes, are among the earliest to respond and among the most studied biomarkers of air pollution [24]. Chlorophyll *a* and *b* absorb light, and carotenoids disperse an excess amount of energy. Excessive energy and reactive oxygen species can also be detoxified using carotenoids. Anthocyanins buffer oxidative loads, while pheophytins detect permanent destruction of chlorophyll, a vital marker of oxidative stress, and are liable to damage from NO_2_ and O_3_, leading to bleaching, reduced photosynthetic efficiency, and injuries [24,25,26,27,28]. However, despite these established facts, in most studies on biomonitoring via pigment responses, it is still treated either as isolated or merged with unrelated traits, so that the recognition of injuries caused by pollution is not eased [28,29,30].

*Ginkgo biloba* has been proven to be well suited for biomonitoring. Its fan-shaped, broad leaves with thick cuticles are efficient in capturing particles, while its vascular and antioxidant systems help it to tolerate stress that would otherwise be harmful to other, more sensitive taxa [9,31]. Despite its apparent resilience, *G. biloba* can exhibit measurable stress responses to chronic pollution long before developing visible symptoms. These responses include variations in the ratios of chlorophylls, carotenoids, and pheophytins [32]. This unique balance of tolerance and sensitivity makes *G. biloba* an effective indicator of urban stress. In addition, it is extensively planted along several urban areas of Europe. Hence, *G. biloba* can provide a stable baseline for multi-year assessments [33]. To evaluate this stress effectively, existing tolerance-based approaches must also be considered.

One widely used composite tool for assessing plant tolerance is the Air Pollution Tolerance Index (APTI) [34], which includes chlorophyll content, ascorbic acid, relative water content, and leaf extract pH [5]. While this tool might be helpful for comparing species, as done in several large-scale surveys [35,36], the problem is that it weights the four variables equally, even though they differ in biological significance. The APTI’s ability to differentiate pigment degradation induced by oxidative processes from the effects of accumulated dust is a major limitation in current bioindicator approaches.

This study tested and introduced the usefulness of the Pigment Integrity-to-Dust Ratio (PIDR) as a novel exploratory biomonitoring index that incorporates two key elements of plant stress: (i) the ratio of chlorophyll to its degradation products (pheophytins) and (ii) the quantity of dust deposited on the leaf surface. The PIDR has not been previously published and is presented here as a conceptual pilot framework. Combining pheophytin with the accumulated foliar dust mass allows the PIDR to link internal biochemical injury with external particulate pressure within a single mechanistic index. It is structured to reflect the spatial gradients across traffic intensities and temporal variations across seasons and years, without the need for normalisation that may mask site-level details. In this work, we present the PIDR as a single-diagnostic framework in a pilot demonstration in Budapest. To examine the effectiveness of the PIDR, we have applied the index to *G. biloba* leaves collected from three sites in Budapest at different times in 2023 and 2024. We hypothesised that (i) the PIDR showed higher spatial contrast in stress than the APTI, (ii) it indicated patterns of changes in plant physiological condition, and (iii) it reflected pollutant-specific patterns.

## 2. Results

### 2.1. Site-Wise Variations in Biochemical Parameters

*Gingko biloba* leaves showed notable physiological and biochemical variations across three sampling locations. MANOVA results indicated significant differences among the control site at Budatétényi Rose Garden, the moderate-traffic site at Dembinszky Street (D), and the high-traffic site at Rákóczi Avenue (R) (F = 5.52, *p* < 0.001) (Table 1). Not all post hoc pairwise contrasts (Tukey’s HSD) were significant between specific sites for individual parameters within trees, where biological variability was high relative to between-site differences. The overall significance achieved demonstrates the pattern’s significance across variables, rather than an overall similarity between individual site pairs. The dust load also differed (F = 3.19, *p* = 0.003), with the high-traffic location again showing the highest loads, demonstrating the effect of increased automobile exhaust emissions. Although it is treated as such, Budatétényi Rose Garden is not exactly a pollution-free habitat; it is merely a low-traffic reference. The control site had, at the time of the September measurements, trees displaying the characteristic values of natural autumnal senescence, not damage from pollution (relative water content in September 2023 at 69.22% and in September 2024 at 70.67%, leaf extract pH in September 2023 at 3.56 and in September 2024 at 3.49). All September values at the control site were interpreted in consequence. Accordingly, both chlorophyll *a* (Chl-*a*) and *b* (Chl-*b*) values of leaves from urban areas, compared to control leaves, were greatly reduced (Chl-*a:* F = 9.29, *p* < 0.001; Chl-*b*: F = 5.55, *p* < 0.001). Chlorophyll levels in 2023 and 2024 were higher at the moderate-traffic site (Chl-*a* = 0.57, Chl-*b* = 0.33) than at the high-traffic site (ΔChl-*a* = −0.20, ΔChl-*b* = −0.13). Notably, the moderate-traffic site in July 2023 recorded the highest level of Chl-*a* (2.09 ± 0.53 mg g^−1^), exceeding both the control (1.95 ± 0.76 mg g^−1^) and the high-traffic site (1.40 ± 0.34 mg g^−1^), contrary to a simple pollution gradient. This likely reflects tree-level physiological heterogeneity and a transient stimulatory chlorophyll response under moderate early-season pollution exposure.

Total chlorophyll was sharply decreased at the moderate-traffic site (Δ = −0.90; F = 7.56, *p* < 0.001), suggesting more severe pigment deterioration with constant exposure. Carotenoid levels were substantially higher at the sites with traffic than in the control site (F = 11.83, *p* < 0.001), although both trafiic sites showed a decline in their levels from 2023 to 2024 (ΔD = −0.10; ΔR = –0.08) (Supplementary Table S1*).* Pheophytin *a* (Pheo-*a*)and *b* (Pheo-*b*) increased at the traffic sites (Pheo-*a*: F = 14.62, Pheo-*b*: F = 5.36, *p* < 0.001), providing evidence of ongoing chlorophyll degradation. There was no temporal change in total pheophytin between 2023 and 2024 at any of the sites. The APTI varied across sites (F = 3.12, *p* = 0.003), with the traffic sites having higher values than the control site. This seemingly counterintuitive result reflects the nature of the APTI as a tolerance-based composite index, i.e., higher APTI values at polluted sites indicate greater physiological tolerance, not lower damage. Trees chronically exposed to pollution may upregulate their ascorbic acid and relative water content as adaptive compensatory responses, thereby increasing their APTI [5]. The moderate-traffic site showed a more pronounced increase in APTI from 2023 to 2024 (Δ = +0.63) than the high-traffic site (Δ = +0.09), suggesting an adaptive response to sustained stress in moderate-traffic conditions.

### 2.2. PIDR

**Figure 1:**
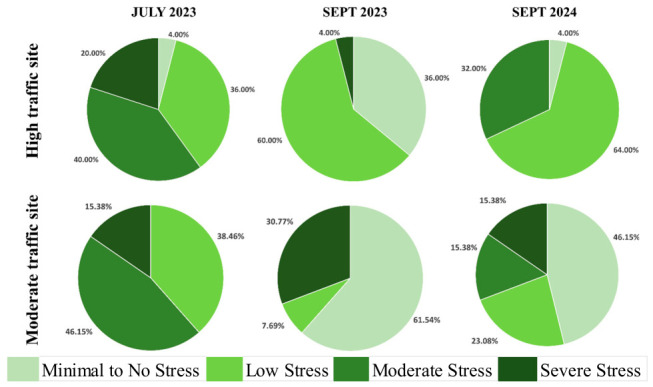
Stress classifications based on *Gingko biloba* leaves from moderate- and high-traffic sites in July 2023, September 2023, and September 2024 as fixed fractions of the asinh-transformed control median. Severe = ≤0.2; moderate = 0.2–0.5; low 0.5–1.0; minimal to no stress = ≥1.0. The size of the segment reflects the percentage of samples in that group, illustrating differences in regional and temporal differences in pigment stability.

Temporal and spatial differences in PIDR-based stress classes were observed between the moderate-traffic and high-traffic sites compared with the control site (Figure 1). PIDR stress-class proportions are summarised at the site level and sampling period (aggregated within individual trees) with the leaves nested within the trees (e.g., R1, R2…), while the spread of individual leaves is reported to illustrate within-site variability. With stress responses ranging from 20% of samples in severely stressed trees to 36% in low-stressed trees and 4% in minimal- to no-stressed trees, the high-traffic site showed a 50% divergence from the control site in July 2023, suggesting a mixed pattern of vulnerability and tolerance. With 46.2% of samples in the moderate-stress class and 38.5% in the low-stress class, the moderate-traffic site showed a more polarised distribution and a slightly greater deviation (53%) than the high-traffic site, indicating less resilience.

By September 2023, a notable divergence had emerged among the sites. The high-traffic site exhibited a 47% deviation from the control site, predominantly influenced by low (60%) classes, indicating a short-term adjustment and partial recovery. Significant variance of 145% was observed at the moderate-traffic site, characterised by a bimodal distribution; 31.0% of samples showed severe stress, and 61.5% showed minimum to no stress. This distribution highlights physiological heterogeneity, likely caused by exposure to microenvironmental dust and pollution. These September 2023 PIDR values should be interpreted with caution because rainfall on 2–3 September 2023 in Budapest washed accumulated summer dust from the leaf surfaces, artificially reducing the dust-load denominator and thereby inflating PIDR at the high-traffic site, rather than signalling genuine biological recovery. Similarly, near-zero Pheo-*a* values at the moderate-traffic site (0.019 mg g^−1^) likely reflected a measurement artefact rather than true physiological condition, as values at or below the detection limit distort the Chl-*a*/Pheo-*a* ratio. The September 2023 data are therefore retained for completeness but should not be used for quantitative temporal comparisons.

At the high-traffic site, the proportion of leaves experiencing minimal to no stress decreased to 4% and shifted towards low (64%) and moderate (32%) stress in September 2024. Leaves showed similar instability across pigments but with little potential for recovery. However, outliers in a mild-manner distribution across all categories are displayed by the mid-traffic site, with an overall 80% change. Approximately 46% of the leaves showed minimal to mild stress, whereas 15% each showed severe or moderate stress.

The moderate-traffic location had more stress signals that did not correlate linearly with traffic intensity, indicating local microenvironmental variation, pollutant and dust exposure, and physiological adjustment dynamics. This site had higher PIDR classes, indicating stress response ranges.

### 2.3. Validation of the PIDR Through Air Pollutants

**Figure 2:**
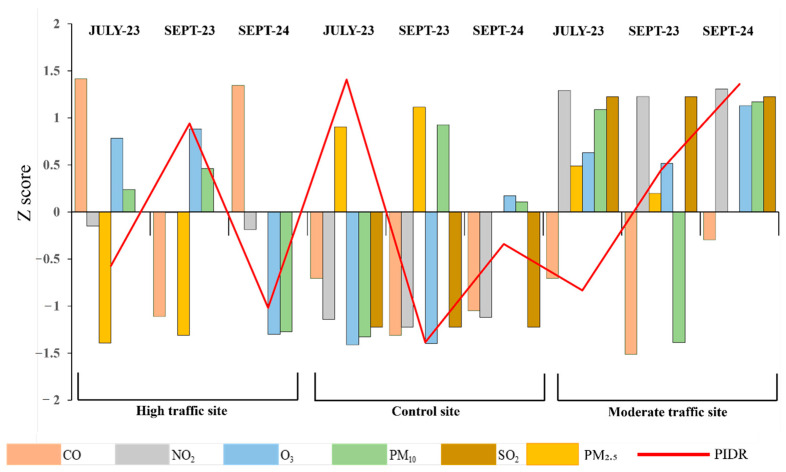
Temporal trends in standardised air pollutant concentrations and Pigment Integrity-to-Dust Ratio (PIDR) across urban areas in Budapest, Hungary. The bar graphs illustrate the Z-scores of significant air pollutants throughout three sites for three time points: July 2023, September 2023, and September 2024. The red line shows site and time-specific PIDR changes.

The PIDR in relation to major air pollutants (CO, NO_2_, O_3_, PM_10_, PM_2.5_, and SO_2_) for three time periods (July 2023, September 2023, and September 2024) is illustrated as a biochemical stress index in Figure 2 and Supplementary Table S2. The control site remained higher across all scenarios, with less pigment degradation at lower pollutant loading. There was a steady drop in PIDR at the control site over time (July 2023 = 1.70, September 2023 = 1.16, September 2024 = 1.08). However, this can be linked to natural seasonal senescence rather than pollutant stress. This decline was driven by a 49% reduction in Chl-*a* (1.95 → 1.30 mg g^−1^ during July → September 2023) due to autumnal senescence and a 127% increase in foliar dust load (3.41 → 7.74 µg cm^−2^), which increased the PIDR denominator independently of pollution-induced pigment degradation. CO remained undetectable at the control site in September 2024, confirming the decrease in PIDR was senescence-driven, not pollution-driven.

The high-traffic site exhibited persistent pigment deterioration, associated with elevated CO and NO_2_ levels. CO rose from 58 ± 15 µg m^−3^ in July 2023 to 328 ± 26 µg m^−3^ in September 2023 (+459%), then declined to 193 ± 17 µg m^−3^ in September 2024. SO_2_ rose sharply from 2.4 ± 0.1 in July 2023 to 25 ± 2 µg m^−3^ in September 2024. Concurrently, NO_2_ at the high-traffic site declined from 27.7 µg m^−3^ in September 2023 to 12.7 µg m^−3^ in September 2024. Therefore, CO and SO_2_, rather than NO_2_, acted as co-dominant stressors, driving the continued decline in PIDR to 0.52.

The moderate-traffic site exhibited modest PIDR gains in September 2024, despite concurrent increases in PM_10_ and PM_2.5_. NO_2_ was higher at the moderate-traffic site in September 2023 (37 ± 2 µg m^−3^) than at the high-traffic site (28 ± 2 µg m^−3^). SO_2_ rose sharply from 4.86 µg m^−3^ in September 2023 to 50.85 µg m^−3^ (+946%) in September 2024, the highest SO_2_ level recorded across all sites, and NO_2_ remained elevated at 25.5 µg m^−3^, together driving PIDR down to 0.11, the lowest recorded across all sites. O_3_ followed an inverse gradient. The control site had the highest O_3_ levels (85 ± 2 µg m^−3^ in July 2023), followed by the moderate-traffic site (77 ± 2 µg m^−3^) and the lowest at the high-traffic site (38 ± 1 µg m^−3^), consistent with NO_2_ titration of O_3_ by traffic-generated NO_2_. By September 2024, O_3_ and NO_2_ at both traffic sites had fallen to undetectable levels due to seasonal photochemical decline and continued NO_2_ titration. The PIDR at the control site is elevated in the Z-score plot (Figure 2) as a normalisation artefact; the anomalous 2.51 PIDR at the moderate-traffic site in September 2023 inflates the Z-score scale and should not be interpreted as a genuine improvement. At the control site, PIDR tracked lower with higher PM_2.5_ (r ≈ −1.00, *p* = 0.007) and higher with increased CO (r ≈ +0.998, *p* = 0.042). At the moderate-traffic site, PIDR declined with higher O_3_ (r ≈ −1.00, *p* = 0.009). At the high-traffic site, PIDR positively correlated with NO_2_ (r ≈ +1.00, *p* = 0.019), whereas it negatively but insignificantly correlated with CO (r = −0.971, *p* = 0.155).

Given these three temporal points and the Z-score normalisation, these near-unit correlations likely inflate effect sizes and should be treated as illustrative signals rather than precise estimates. Multiple regression, therefore, serves as a screening step, indicating CO as the strongest negative predictor at the high-traffic site (β = −0.80) and, likewise, PM_2.5_ at the control site (β = −1.00) and O_3_ at the moderate-traffic site (β = −1.00) (Supplementary Table S3).

## 3. Discussion

This study suggests that the PIDR has strong potential to detect spatial and temporal gradients in physiological stress in *G. biloba* across an urban ecosystem with different traffic levels, from low traffic (Budatétény Rose Garden) to moderate traffic (Dembinszky Street) and high traffic (Rákóczi Avenue), paralleling measured pollutant loads. The chlorophyll-to-pheophytin ratio reveals pigment breakdown in leaves. The PIDR interprets this change as a response to dust buildup, turning the physiological measurement into an indicator of particulate exposure. These patterns are consistent with previous findings that particulate deposition compromises pigment integrity in roadside trees under chronic dust exposure [37], as observed in clear urban–rural dust gradients in Vienna [38]. The researchers in [39] found that trace-metal-rich urban dust altered pigment composition and leaf functional traits in *Tilia cordata*. Thus, the PIDR’s rationale for integrating particulate accumulation with pigment degradation into a single mechanistic index is recommended. Mechanistically, the PIDR is consistent with both oxidative and mechanical stress pathways. Although dust particles themselves are not direct biochemical toxins, their deposition on leaf surfaces reduces the irradiance reaching the chloroplasts, blocks stomata, restricts gaseous exchange, and increases leaf surface temperature, and the particles can carry phytotoxic trace metals that induce oxidative stress [37]. Gaseous pollutants such as O_3_ and NO_2_ accelerate chlorophyll demetallation and pheophytin accumulation [40,41,42] and mechanical stress, where dust deposition reduces irradiance, obstructs stomata, and amplifies localised oxidative reactions [37,43,44,45].

Compared to the APTI, the ratio of chlorophyll and pheophytin in the PIDR, along with the external dust load, is a more effective indicator of plant internal damage. This disparity in the temporal analysis is notably evident from September 2023 to September 2024, during which the PIDR tracked both a decrease and a partial recovery in plant health, whereas the APTI failed to detect them. Moreover, the PIDR showed specificity for particular pollutants, consistent with findings from other European studies demonstrating a negative relationship between O_3_ levels and chlorophyll degradation and pheophytin formation in response to moderate traffic [40,41]. Also, in high-traffic sites, a reduction in the PIDR correlated with increased NO_2_ concentration, reflecting that NO_2_ limited photosynthesis and altered foliar biochemical processes [46,47].

SO_2_ negatively affects chlorophyll [48] by inducing degradation via magnesium loss [48]. This was reflected in the simultaneous reduction in Chl-*a* and Chl-*b* and an increase in pheophytin, suggesting that reactive oxygen species facilitated pigment damage [49,50]. Additionally, increased levels of carotenoids suggested a photoprotective response in plants [51]. Together, these mechanisms can explain pigment decline at the high-traffic site, the fluctuating yet recovering pattern observed at the moderate-traffic site, and the overall stability in the control site. Seasonal PIDR patterns supported this interpretation that the moderate-traffic site showed wide fluctuations in 2023 but settled into a more stable, lower-stress condition by 2024, hence reflecting partial recovery. These shifts either revealed improvement in air quality or adaptive responses in *G. biloba*. Similar seasonal patterns in pigment composition and particulate matter deposition have been reported in several other urban tree species, further supporting our findings [52,53,54].

As indicated by [55,56], the sharp decline in the PIDR at high-traffic locations might reflect environmental stress within the urban ecosystem, an outcome of chronic pollutant exposure amplified by the urban heat island. These patterns must be treated purely as exploratory rather than as conclusions, given the short time frame covered by our study. In future analyses, more samples should be collected per station and per season, and fixed- and mixed-effects models should be used to examine the addition of weather data.

A methodological disadvantage of the PIDR must be mentioned. If the pheophytin concentration is close to zero, the chlorophyll: pheophytin ratio might be disproportionately high, making PIDR levels appear high regardless of the pollution load. This might limit the application of the PIDR to samples taken early in the season or at clean sites, and demand its recalibration for species, climatic zone, or urban conditions, rather than its random use.

The variables in the APTI are pH, relative water content in the leaf, and ascorbic acid and chlorophyll levels [5], whose combination of parameters could be useful to establish general tolerance categories but may not be as sensitive as to capture the pattern of pollutant-specific responses since such components are driven by the variations in micro-climatic variables [44]. The APTI measures a plant’s tolerance to pollution; more resistant plants have a higher APTI, while more susceptible plants have a lower APTI. Since it includes different types of plant attributes, the APTI measures all of them. The PIDR is directly linked to pigment loss caused by dust deposition on leaves and is more closely related to the plant stress index. Hence, the APTI and PIDR cannot be substituted but need to complement each other. A low APTI at a low-traffic site does not necessarily mean the plant sustained more damage, because the APTI accounts for many factors. A small increase in the APTI at polluted sites may indicate that the plant is adapting, but it does not necessarily mean it is under less stress. When we compared the APTI and PIDR, the PIDR matched more closely with the chemicals causing pigment loss from pollution. The PIDR, unlike the APTI, monitors both chlorophyll and its breakdown product, pheophytin, and accounts directly for dust deposition on the leaf. These differences highlight the methodological differences between the indices and explain why they evaluate pollution impacts differently [37,40,57,58,59]. Based on our results, the two indices can be considered complementary; the APTI can be useful for ranking species-level tolerance, while the PIDR can provide a more detailed diagnostic for pollutant pathways.

The results from Budapest are consistent across Europe regarding pigment loss and accumulation due to traffic dust. Particulate matter accumulation varied with traffic intensity and leaf wax characteristics in Vienna [60], while pigment impairment after exposure to particulate matter was noted in *Betula pendula*, *Quercus robur*, and *Tilia cordata* in Warsaw [61]. Therefore, while the APTI remains important for species-tolerant ranking, the PIDR provides mechanistic reasoning for why pigments degrade under particulate loads at the site level. Examples like these strengthen the need for an index that combines biochemical stress and particulates, the two causes of degradation. However, the PIDR thresholds were tested at the local level and need recalibration for other tree species in different locations. Incorporation of Receiver Operating Characteristic (ROC)-derived cut-points or a classification algorithm would have improved the solidity and transferability of these thresholds. The concentrations of pollutants vary from year to year due to changes in weather, discharges, and ecological factors. Year-to-year pollutant concentrations vary due to meteorological factors (temperature inversions, wind speed and direction, and precipitation patterns), changes in local emissions from traffic and residential heating, and urban topology that affects pollutant dispersion. Consequently, these pollutants diminish chlorophyll activity by inhibiting photosynthesis and causing oxidative stress, reducing molecules to tiny remnants that may destabilise pigments. However, the data loss makes it difficult to link pollutants directly to the PIDR. Since the chlorophyll:pheophytin ratio is not always distinct, especially when pigment levels are very low, the PIDR’s lack of reliability underscores the need to recalibrate in each area to achieve accurate results. Furthermore, it is noteworthy that the PIDR serves as a time-integrating bioindicator: leaf pigment ratios and foliar dust loads reflect the physiological stress imposed on the leaf over preceding weeks more than instantaneous pollutant concentrations at the time of sampling. The pollutant substance itself does not accumulate within the tissue; the biological response—ongoing pigment degradation and gradual dust deposition—integrates the history of exposure. The three sampling windows (July 2023, September 2023, September 2024) were therefore chosen to represent seasonal and interannual contrasts, and the air pollutant data were used as period-averaged contextual values rather than a continuous record. Higher-frequency monitoring paired with meteorological covariates would therefore further refine the pollutant–PIDR relationship in future work.

While the high-traffic site was under repetitive and chronic stress with limited recovery, the moderate-traffic site swayed seasonally under extreme vulnerability and intermittent levels of recovery, until 2024 when it stabilised. This reflects higher dust levels on roads and impairment of pigment characteristics near traffic corridors, as reported in Europe. Fluctuations relative to the control site were driven by other seasonal factors, including heat stress, photodegradation, and particle deposition. Particulate loads varied with traffic levels and leaf wax signatures in Vienna [60]. In Warsaw, certain species, such as *B. pendula*, *Q. robur*, and *T. cordata*, showed colour degradation following exposure to particulate matter [61]. The findings from Vienna and Warsaw together suggest that the APTI continues to be able to help rank species. The PIDR may help identify a potential link between an external driver (particulate deposition) and internal pigment damage, which is essentially what the PIDR measures. Thus, the indices are complementary and necessarily helpful for assessing oxidative stress and particulate accumulation in urban trees. Calibrating the PIDR anew might be required for other tree species and locations. Threshold determination, for instance, using ROC curves or classification algorithms, is crucial in this direction of generalisation across tree species and cities as well.

While *G. biloba* is tolerant of pollution and drought, other tree species may exhibit greater pigment loss under urban conditions, as shown for *B. pendula*, *Q. robur*, and *T. cordata* [61]. Heavy dust deposition altered leaf optical properties, accompanied by a decline in chlorophyll concentration and Specific Leaf Area (SLA) [62]. Assessing species with contrasting leaf morphologies and sensitivities would allow the PIDR applicability range to define the generality of the PIDR response. To refine the pollutant–PIDR links, adding even more variables, e.g., meteorological factors, to the mixed-effect models would further help clarify the background signal. Nevertheless, preliminary results suggest the PIDR is useful for assessing urban pollutant impacts and that it provides a more direct interpretation than APTI results. The PIDR may therefore help advance beyond the results of current bioindicators [35] in today’s cities, which reflect the multifaceted stresses within them [61,62]. The PIDR may have potential beyond scientific questions, helping cities with their urban planning and the selection of air quality policies [63].

## 4. Materials and Methods

### 4.1. Study Sites and Sample Collection

**Figure 3:**
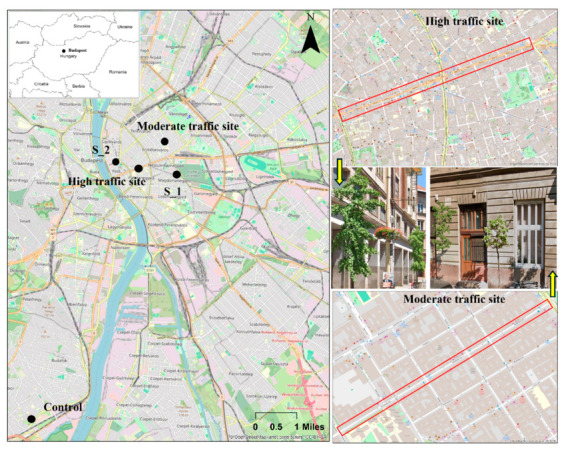
Map of the study area in Budapest, Hungary, showing sampling and monitoring sites. The three sites for leaf collections were Budatétényi Rose Garden (control site (C)), Dembinszky Street (moderate traffic), and Rákóczi Avenue (high, dense traffic). Measurement values of atmospheric CO, NO_2_, O_3_, PM_10_, PM_2.5_, and SO_2_ were derived from the National Air Quality Monitoring Network (stations in Budatétény Rose Garden as background values (C), Teleki Square (S_1), and Erzsébet Square (S_2).

The study was carried out by collecting samples from *G. biloba* trees at three sites in Budapest (Figure 3). Each site reflected a distinct degree of traffic intensity: Rákóczi Avenue (1.56 km long, 35 m wide), characterised by high traffic; Dembinszky Street (0.76 km long, 17 m wide), with moderate traffic; and Budatétény Rose Garden, which functioned as a urban reference, was a control site with low traffic, selected for its minimal direct vehicle emission exposure relative to the other two sites.

The sampled *G. biloba* trees were established as field-grown roadside trees located directly along the roadside footway adjacent (approximately 1–3 m from the road edge) to the traffic corridor. Leaves were collected in two seasons, July and September 2023, and September 2024, to examine annual variation. Ten healthy *G. biloba* trees were sampled at each location. Ten fully developed leaves, approximately 2 m above ground, were harvested from each tree’s traffic-facing side, following the microenvironment exposure standardisation protocol. For each tree, five fully developed leaf samples were used to perform pigment extractions, with three replicate extracts measured. Since sample collection at the tree level is crucial for independent results and to avoid pseudoreplicates, all samples were collected on a rainfall-free day. The leaves were immediately flash-frozen with liquid nitrogen on site, then stored at −70 °C for subsequent biochemical extractions. Air pollution measurements from stations in Teleki Square (moderate traffic) and Erzsébet Square (high traffic) were obtained from their official websites. Air pollution data for the control site were retrieved from the nearest local suburban area, Budapest Budatetenyi, due to a lack of a device at the site.

### 4.2. Deposited Dust Analysis

Leaf area was determined by scanning each leaf and using ImageJ software (Version 1.52a, Wayne Rasband, National Institute of Health, USA). Each leaf was then washed in 250 mL of deionised water for 10 min on an orbital shaker to dislodge the adsorbed particulate matter, followed by a second rinsing with 50 mL to ensure thorough recovery. The pooled 300 mL suspension was passed through a 150 µm mesh sieve/screen to remove larger debris, after which the filtrate was subjected to vacuum filtration using preweighed filter papers (5–8 µm retention; Munktell 392, Ahlstrom, Espoo, Finland). Filters were then dried to constant weight (±0.1 mg/±1 mg) for gravimetric determination of dust, following rigour in mass-difference protocols [63]. Dust accumulation was normalised against leaf surface area and expressed as µg cm^−2^ [64].

### 4.3. Pigment Extraction

Fresh leaves (20 mg fresh mass) were homogenised by means of a grinding apparatus (2500 rpm) in 5 mL of 96% ethanol for 4 min. Subsequently, the extract was centrifuged, and the upper liquid phase was stored in the dark at 4 °C. A UV/VIS spectrophotometer (BOECO S220) measured the absorbance of the cleared extract in a 1 cm quartz cell in the presence of absolute ethanol. To determine the pheophytin concentration, the same ethanolic extract used previously [65] was used. Absorbance readings were recorded prior to acidification at wavelengths 649, 665, and 750 nm. One drop (~50 µL) of 10 M HCl (final concentration of approximately 0.1 M HCl) was introduced, and the pheophytin was generated by removing Mg^2+^ from all the chlorophyll. After 3 min, the absorbances were remeasured at wavelengths 655, 666, and 750 nm. All operations were carried out under reduced light conditions to avoid photodegradation of the pigments. For statistical analyses, replicate values were averaged at the tree level, with each tree treated as a biological replicate.

Equations (1)–(4) were used to calculate the chlorophyll and carotenoid concentrations in mg g^−1^ fresh mass.

(1) $$C_{\text{Chl-}a}=\left[13.36\left(A665-A750\right)-5.19\left(A649-A750\right)\right]\times \frac{0.005}{m}$$

(2) $$C_{\text{Chl-}b}=\left[27.43\left(A649-A750\right)-8.12\left(A665-A750\right)\right]\times \frac{0.005}{m}$$

(3) $$\mathrm{Total}\;\mathrm{chlorophyll}=[5.24(A665-A750)+22.24(A649-A750)]\times \frac{0.005}{m}$$

(4) $$C_{\mathrm{car}}=\frac{1000\;\left[A470-A750\right]-2.13\;C_{\text{Chl-}a}-97.64\;C_{\text{Chl-}b}}{209}\times \frac{0.005}{m}$$

where C_Chl-*a*_ = the concentration of Chl-*a*, C_Chl*-b*_ = the concentration of Chl-*b*, C_car_ = the concentration of carotenoids, A665 − A750 = the absorbance at 665 nm minus the absorbance at 750 nm, A649 − A750 = the absorbance at 649 nm minus the absorbance at 750 nm, A470 − A750 = the absorbance at 470 nm minus the absorbance at 750 nm and m = the fresh mass of the sample in grams. Equations (5)–(7) were used to calculate the levels of Pheo-*a*, Pheo-*b*, and total pheophytin in mg g^−1^ fresh mass [66].

(5) $$\text{Pheo-}a=\left[\left(20.15\;\left[A_{666}-A_{750}\right]-5.87\;\left[A_{655}-A_{750}\right]\right)-\left(11.63\;\left[A_{665}-A_{750}\right]-2.39\;\left[A_{649}-A_{750}\right]\right)\right]\times \frac{0.005}{m}$$

(6) $$\text{Pheo-}b=\left[\left(31.9\;\left[A_{655}-A_{750}\right]-13.4\;\left[A_{666}-A_{750}\right]\right)-\left(20.11\;\left[A_{649}-A_{750}\right]-5.18\;\left[A_{665}-A_{750}\right]\right)\right]\times \frac{0.005}{m}$$

(7) $$\mathrm{Total}\;\mathrm{pheophytin}=\left[\left(6.75\;\left[A_{666}-A_{750}\right]+26.03\;\left[A_{655}-A_{750}\right]\right)-\left(6.45\;\left[A_{665}-A_{750}\right]+17.72\;\left[A_{649}-A_{750}\right]\right)\right]\times \frac{0.005}{m}$$

where A_666−750_ = the absorbance at 666 nm minus the absorbance at 750 nm, A_655−750_ = the absorbance at 655 nm minus the absorbance at 750 nm, A_665−750_ = the absorbance at 665 nm minus the absorbance at 750 nm, A_649−750_ = the absorbance at 649 nm minus the absorbance at 750 nm, and m = the fresh mass of the sample in grams.

### 4.4. APTI

**Figure 4:**
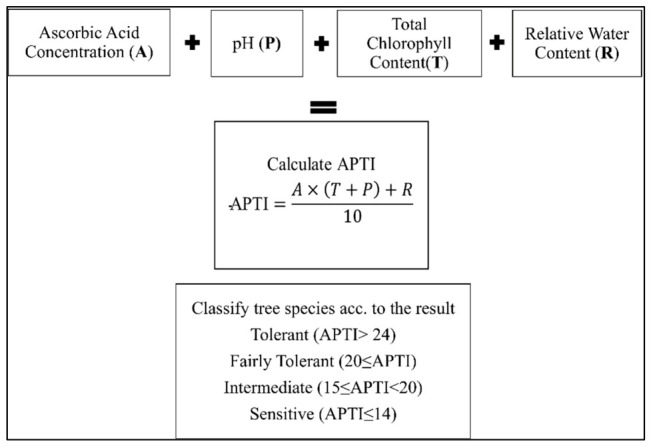
Parameters from calculation of the Air Pollution Tolerance Index [34].

The APTI was calculated based on the concentrations of chlorophyll and ascorbic acid and relative water content in the leaves, as well as the pH of leaf extracts (Figure 4) [5,67].

### 4.5. PIDR Calculation for G. biloba

The PIDR combines two diagnostic aspects of leaf stress: (i) the balance between intact chlorophyll pigments (Chl-*a* and Chl-*b*) and their degradation products (Pheo-*a* and Pheo-*b*) and (ii) the accumulation of dust on the leaf surface. The process of averaging the two pigment ratios balances the index while reducing the risk of bias from variations in any individual pigment. Pheophytin is a result of chlorophyll hydrolysis during leaf senescence or due to pollution stress, not an artefact of acidification. Leaves synthesise pheophytin during leaf senescence, following the degradation sequence chlorophyll > pheophytin > pheophorbide. The spectrophotometer readings collected before acidification thus reflect chlorophyll degraded to pheophytin naturally under field conditions. After acidification, the spectrophotometer measures the total chlorophyll present, since acidification converts the remaining intact chlorophyll to pheophytin. The chlorophyll:pheophytin ratio computed from these two sets of measurements shows the quantity of intact chlorophyll that is present relative to the chlorophyll degraded to pheophytin and can serve as a measure of degraded chlorophyll under the given stresses. Adding dust load to the denominator gives the PIDR, which measures pigment health compared to the amount of dust on the leaf. This connects changes in leaf pigment to dust buildup. Equation (8) was used to calculate the PIDR for *G. biloba* and morphologically similar broad-leaved species.

(8) $$PIDR\;of\;G.biloba=\frac{\left(\frac{Chl-a}{Pheo-a}+\frac{Chl-b}{Pheo-b}\right)}{2\times \mathrm{dust}\;\mathrm{load}\;(\mu {g/\mathrm{cm}}^{-2})}$$

The values of the Chl-*a*, Chl-*b*, Pheo-*a*, and Pheo-*b* concentrations were given as mg g^−1^ fresh weight. The numerator and denominator in pigment-ratio computations had the same units; therefore, the ratios Chl-*a*/Pheo-*a* and Chl-*b*/Pheo-*b* are dimensionless. With pollution, when all chlorophyll is broken down into pheophytin, the numerator (Chl-*a*/Pheo-*a* + Chl-*b*/Pheo-*b*) would theoretically approach 0; under mild or prolonged pollution stress, it would rise to well above 2.0 in leaves from low-pollution sites where chlorophyll far exceeds pheophytin. Dust load was the gravimetric measurement of dust particles divided by the leaf surface area. The PIDR has units of the dust load denominator, but in this study, the PIDR is expressed as a fold change relative to the mean PIDR of high-traffic-exposed sites, compared with the median PIDR of the low-traffic reference site during the same period, making the final index dimensionless. The averaging of the two pigment ratios also normalises the index and reduces the risk that any single pigment disproportionately influences it.

#### 4.5.1. Site-Level Deviation Analysis

The PIDR values were presented as a fold change relative to the control site’s median (Mt) (Equation (9)) for each sampling date to enable comparisons among sites.

(9) $${\mathrm{Fold}\;\mathrm{Change}}_{i,t}=PIDRi,t/Mt$$

where PIDR refers to the raw value for tree i at time t. Site-level deviations were then summarised for each site by using the mean ± SD) and median ± median deviation from the control site (MDC). TheMDC was calculated as:

(10) $$MDC=\mathrm{median}\left(\left|{\mathrm{FoldChange}}_{i,t}-\mathrm{Median}\left({\mathrm{FoldChange}}_{t}\right)\right|\right)$$

where i = individual tree sample, t = sampling date, *Fold Change_i,t_* = PIDR of tree i divided by the control site’s median PIDR for date t, and Median *(Fold Change)ₜ* = the median fold change for that date or site. The mean ± SD and median ± MDC were converted into percentage deviation from the control baseline to aid comparison across sites:

(11) $$\%\mathrm{MDCt}\;=\;100\;\times \;\mathrm{median}\;\left(\left|{\mathrm{FoldChange}}_{i,t}-\mathrm{Median}\left({\mathrm{FoldChange}}_{t}\right)\right|\right)$$

The value refers to the median of the fold change distribution for a given site. The SD and MDC were similarly scaled by multiplying by 100. While mean ± SD values were retained for comparison, the primary summaries emphasised the median ± MDC due to their robustness against outliers and better representation of the central tendency and spread within each site. This approach ensured that ecological interpretation was not disproportionately influenced by a few extreme individuals while still capturing the dominant stress patterns across sites.

#### 4.5.2. Stress Classification

To create a stress classification, limits were set relative to the control site to maintain consistency across sites and dates. The median PIDR at the control site (MC) was used as a no-stress standard for every sampling period, rather than a single fixed pigment ratio. The transformed control value is referred to as the baseline. PIDR values, which do not have a normal distribution, were adjusted by an inverse hyperbolic sine (asinh) transformation to make it easier to compare fold changes. For interpreting the entire PIDR range, a uniform analytical scale was used. The response is stable for values closer to zero and helps identify differences among larger values, lessening the impact of extreme values. Also, the asinh transformation fits ecological response patterns, showing gradual changes at lower pollution levels and sharper responses at higher stress levels.

Each individual measurement (PIDRi) was expressed as its deviation from the baseline:

(12) Δi=Asinh(PIDRi)−B

where Δi = the difference from the baseline, PIDRi = raw PIDR value for sample i, and B = the baseline. A positive Δi means the leaf is close to or above the control (healthier), while a negative Δi means the leaf has lower pigment integrity (more stress).

The MC was multiplied by fixed fractions (f = 1.0, 0.5, and 0.2) corresponding to varying levels of pigment loss to establish stress thresholds. To ensure that all threshold values were comparable and expressed on the same scale, these scaled values were subsequently transformed using the same inverse hyperbolic sine (asinh) transformation as the sample data.

(13) Tf=asinh(f×MC)−B

where Tf = transformed threshold for each level. Accordingly, stress categories were defined as minimal to no stress = Δi ≥ T_1.0_, low stress = T_0.50_ < Δi < T_1.0_, moderate stress = T_0.20_ < Δi ≤ T_0.50_; and severe stress = Δi ≤ T_0.20_.

To offer a paradigm that is both replicable and ecologically interpretable, stress thresholds were specified as fixed fractions of the MC. Minimal- to no-stress samples were defined as at least as high as the control (≥1.0 × MC), which is the physiological baseline for healthy foliage. To capture slight deteriorations in pigment integrity that signify early physiological compensation while being above the functional midpoint, the range 0.50–<1.0 × MC was consider low stress. Studies by [68,69,70] indicate that a 50% decline in fluorescence or chlorophyll is often treated as a meaningful decline but not yet a severe level of physiological loss. For this reason, we used 0.50 (50% of MC) to mark moderate stress. Severe stress was defined with a lower cut-off of 0.20 (20% of MC), reflecting findings that a 20–30% drop is typically associated with major damage to pigment pools and photosynthetic function [69,71]. In addition to providing a repeatable, ecologically significant classification across sites and dates, this proportional fold-of-control paradigm explicitly links stress intensity to pigment integrity loss relative to the low–traffic urban reference baseline.

### 4.6. Statistical Analysis

Statistical analyses were performed using IBM SPSS Statistics (version 21). A multivariate analysis of variance (MANOVA) was applied to analyse the overall effect of site on Chl-*a*, Chl-*b*, total chlorophyll, carotenoids, pheophytin, and dust deposition. One-way ANOVAs and Tukey’s Honestly Significant Difference test were used to examine pairwise contrasts across three sites during the years when statistical differences were detected by the MANOVA; where Tukey’s HSD yields no significant pairwise contrast for a specific parameter, shared superscript letters are correctly assigned even when the omnibus test is significant, reflecting the conservative familywise correction and the biological variability inherent among field-grown urban trees. Levene’s test was used to assess homogeneity of variances, and a one-way ANOVA was employed to analyse temporal variation across the three sampling dates, since data for each site were not collected from the same tree repeatedly. Comparisons among leaves collected on one date from the same plant were within each tree section, not from all parts of the tree.

Pearson’s correlation coefficients, based on standardised PIDR, were computed to determine the relationships between pollutant concentrations and the PIDR at each site. For each site, separate multiple regression equations were developed, with the PIDR as the dependent variable, to identify the most influential pollutant that accounted for the greatest variation in PIDR. The relatively large standard deviations observed for some parameters reflect the biological heterogeneity expected among field-grown urban trees exposed to variable local environments. The two strongest predictors were used in every case: for the high-traffic site, CO and PM_2.5_; for the control site, O_3_ and PM_2.5_; and for the moderate-traffic site, O_3_ and PM_2.5_. To compare predictor strength, standardised coefficients (β) were used, and model fit was evaluated with R^2^. Before interpreting model output, residual normality and collinearity (Variance Inflation Factor < 5) were validated. Because of the limited number of sampling dates, the regression analyses were interpreted as exploratory rather than definitive.

## 5. Conclusions

The PIDR is an effective measure of pollution stress in *G. biloba*. Based on our data, the greater the dust accumulation on leaves, the lower the PIDR value, resulting in greater chlorophyll loss and a higher level of pheophytin. The PIDR of leaves in a site away from dust accumulation increased. This followed well-known physiological pathways in which carbon in the city could suppress photosynthesis, leading to stomatal closure, surface abrasion, and hindered photo flux to leaves [72]. Also, with increased O_3_ and other pollutants, such as peroxyl radicals, chlorophyll degradation accelerates, and pheophytin production accelerates. This demonstrates a direct link between the integrity of photosynthetic pigments and the accumulation of air particulates. Thresholds should be locally recalibrated. Recent research [73] suggests that, given varying climate zones and cities, indices should be calibrated locally for more sensitive plant species.

Temporal resolution can be improved by adding a single sampling in July, thereby enhancing the assessment of interannual and seasonal variation in the dataset. One sample a year may not accurately reflect the overall picture, as it could miss sudden short-term variation that occurs over a few days. Future studies should improve seasonal monitoring and take samples more regularly throughout the year, from early development to senescence, such as every two weeks or monthly, which might improve PIDR precision and efficient evaluation.

For PIDR to function effectively and efficiently, it should be coupled with biochemical indices, such as metabolites and antioxidants. Datasets derived from PIDR could also improve forecasting when coupled with models like microscale dispersion models and urban climate models. At broader scales, remote sensing-derived metrics, such as the Normalised Difference Vegetation Index, Photochemical Reflectance Index, and Green Normalised Difference Vegetation Index, can extend assessments from the leaf to the canopy level. Developing a portable instrument for pigment and dust measurements could make real-time monitoring a field-based citizen science effort. Together, these efforts would support PIDR’s evolution from a local diagnostic tool to a flexible framework that links plant physiology with urban architecture and policy planning.

## Figures and Tables

**Table 1:** Air Pollution Tolerance Indices and pigment concentrations (mean ± SD) and analysis of variance results at three different sites in Budapest, Hungary. Different superscript letters within the same sampling period indicate significant differences among sites according to Tukey’s Honestly Significant Difference test (*p* < 0.05). Shared superscript letters indicate non-significant pairwise differences under Tukey’s HSD; same-letter assignments are consistent with a significant omnibus MANOVA where within-tree biological variability attenuates individual pairwise contrasts.

Parameter	Sampling Period	Studied Sites			Results of ANOVA	
		High Traffic	Moderate Traffic	Control	F	*p*
APTI	July 2023	13.18 ± 1.46 ^a^	12.39 ± 1.73 ^a^	11.40 ± 1.34 ^a^	3.12	0.003
	September 2023	12.62 ± 1.32 ^a^	11.37 ± 3.77 ^ab^	8.73 ± 6. 10 ^b^		
	September 2024	12.15 ± 1.72 ^a^	11.44 ± 3.78 ^ab^	8.16 ± 5.56 ^b^		
Carotenoid, mg g^−1^ FM	July 2023	0.38 ± 0.07 ^a^	0.48 ± 0.07 ^a^	0.42 ± 0.14 ^a^	11.83	<0.001
	September 2023	0.32 ± 0.05 ^ab^	0.37 ± 0.12 ^a^	0.22 ± 0.16 ^b^		
	September 2024	0.25 ± 0.04 ^a^	0.28 ± 0.14 ^a^	0.23 ± 0.16 ^a^		
Chlorophyll *a*, mg g^−1^ FM	July 2023	1.40 ± 0.34 ^a^	2.09 ± 0.53 ^b^	1.95 ± 0.76 ^ab^	9.29	<0.001
	September 2023	1.24 ± 0.27 ^ab^	1.79 ± 0.65 ^b^	0.99 ± 0.76 ^a^		
	September 2024	1.02 ± 0.27 ^a^	1.20 ± 0.63 ^a^	0.97 ± 0.77 ^a^		
Chlorophyll *b*, mg g^−1^ FM	July 2023	0.77 ± 0.19 ^a^	1.21 ± 0.36 ^a^	1.17 ± 0.50 ^a^	5.55	<0.001
	September 2023	0.72 ± 0.21 ^ab^	1.11 ± 0.43 ^b^	0.62 ± 0.50 ^a^		
	September 2024	0.70 ± 0.21 ^a^	0.89 ± 0.46 ^a^	0.73 ± 0.57 ^a^		
Total Chlorophyll, mg g^−1^ FM	July 2023	2.17 ± 0.53 ^a^	3.30 ± 0.88 ^b^	3.13 ± 1.25 ^ab^	7.56	<0.001
	September 2023	1.96 ± 0.43 ^ab^	2.90 ± 1.08 ^b^	1.62 ± 1.26 ^a^		
	September 2024	1.72 ± 0.48 ^a^	2.08 ± 1.09 ^a^	1.70 ± 1.33 ^a^		
Pheophytin *a*, mg g^−1^ FM	July 2023	0.19 ± 0.05 ^a^	0.24 ± 0.08 ^a^	0.25 ± 0.11 ^a^	14.62	<0.001
	September 2023	0.11 ± 0.07 ^a^	0.02 ± 0.06 ^a^	0.10 ± 0.10 ^a^		
	September 2024	0.13 ± 0.03 ^a^	0.07 ± 0.08 ^a^	0.11 ± 0.10 ^a^		
Pheophytin *b*, mg g^−1^ FM	July 2023	0.39 ± 0.08 ^a^	0.50 ± 0.13 ^a^	0.50 ± 0.23 ^a^	5.36	<0.001
	September 2023	0.34 ± 0.07 ^a^	0.35 ± 0.14 ^a^	0.30 ± 0.25 ^a^		
	September 2024	0.30 ± 0.05 ^a^	0.28 ± 0.15 ^a^	0.31 ± 0.23 ^a^		
Total Pheophytin mg g^−1^ FM	July 2023	0.58 ± 0.13 ^a^	0.74 ± 0.21 ^a^	0.74 ± 0.34 ^a^	8.06	<0.001
	September 2023	0.46 ± 0.12 ^a^	0.37 ± 0.18 ^a^	0.40 ± 0.34 ^a^		
	September 2024	0.43 ± 0.08 ^a^	0.35 ± 0.22 ^a^	0.41 ± 0.33 ^a^		

## Data Availability

Data will be made available on request.

## Supplementary Materials

The following supporting information can be downloaded at: https://www.mdpi.com/article/10.3390/plants15121893/s1, Table S1: Biochemical parameters and pigments across zones, with mean differences relative to the control and temporal changes between 2023 and 2024; Table S2: Summary of air pollution concentration (mean ± SE) of the studied month in the studied sites, Table S3: Z-scores of significant air pollutants and PIDR throughout zones in different sites for three temporal intervals: July 2023, September 2023, and September 2024.
