# Supplementary material for: Pigment Integrity-to-Dust Ratio (PIDR): A Novel Bioindicator for Assessing Urban Air Pollution Stress in Ginkgo biloba
Source: Plants (Basel). 2026 Jun 18;15(12):1893. doi: 10.3390/plants15121893 (PMC13306462; doi:10.3390/plants15121893)
Supplement: Supplementary file 1 [file plants-15-01893-s001.zip › plants-4225980-supplementary.pdf]

Supplementary Table S1. Biochemical parameters and pigments across zones, with mean differences relative to the control (Eq. 2) and temporal changes between 2023 and 2024 (Eq. 1). $\Delta R \,\&\, \Delta D = \overline{X}2024 - \overline{X}2023$ (Equation 1) and $\mathrm{Zone} - \mathrm{Control} = \overline{X}\mathrm{Zone} - \overline{X}\mathrm{Control}$ (Equation 2)

Parameters	R–Control (2023)	R–Control (2024)	ΔR (2024–2023)	D–Control (2023)	D–Control (2024)	ΔD (2024–2023)	F	P
APTI	3.9	3.99	0.09	2.64	3.28	**0.63**	3.12	0.003
Carotenoids	0.1	0.02	-0.08	0.15	0.05	-0.1	11.83	<0.001
Chl-a	0.25	0.05	-0.2	0.8	0.23	**-0.57**	9.29	<0.001
Chl-b	0.11	-0.03	-0.13	0.49	0.16	**-0.33**	5.55	<0.001
Total Chl	0.34	0.02	-0.32	1.29	0.39	**-0.9**	7.56	<0.001
Pheo-a	0.1	0.1	0.01	0.03	0.04	0.02	14.62	<0.001
Pheo-b	0.04	0.04	0.01	0.02	0.01	0	5.36	<0.001
Pheophytin	0.14	0.14	0	0.05	0.05	0	8.06	<0.001

Supplementary Table S2. Summary of air pollution concentration (mean ± SE) of the studied month in the studied sites.

Studied sites	Month	CO (µg/m³)	NO_2 (µg/m³)	O_3 (µg/m³)	PM_{10} (µg/m³)	$PM_{2.5}$ (µg/m³)	SO_2 (µg/m³)
Control	Jul-2023	< LoD	14 ± 1	85 ± 2	13 ± 1	6.6 ± 0.4	< LoD
Control	Sep-2023	< LoD	19 ± 1	67 ± 3	19 ± 2	8.4 ± 0.6	< LoD
Control	Sep-2024	< LoD	4.9 ± 2	< LoD	17 ± 2	7.0 ± 0.6	< LoD
Moderate Traffic	Jul-2023	< LoD	24 ± 1	77 ± 2	19 ± 1	9.1 ± 0.7	4.9 ± 0.1
Moderate Traffic	Sep-2023	209 ± 52	37 ± 2	51 ± 2	24 ± 2	6.4 ± 1.1	5.3 ± 0.3
Moderate Traffic	Sep-2024	63 ± 35	25 ± 2	< LoD	21 ± 3	8.8 ± 1	50 ± 5
High Traffic	Jul-2023	58 ± 15	18 ± 1	38 ± 1	19 ± 1	8.2 ± 0.5	2.4 ± 0.1
High Traffic	Sep-2023	328 ± 26	28 ± 2	25 ± 1	25 ± 2	8.1 ± 1	2.6 ± 0.2
High Traffic	Sep-2024	193 ± 17	13 ± 1	< LoD	11 ± 1	4.4 ± 0.5	25 ± 2

.

Supplementary Table S3. Z-scores of significant air pollutants and PIDR throughout zones in different sites for three temporal intervals: July 2023, September 2023, and September 2024.

**Sample**	**Month**	**Z CO**	**Z NO_2**	**Z O_3**	**Z PM_{10}**	**Z $PM_{2.5}$**	**Z SO_2**	**Z PIDR**
	Jul-23	1.4	-0.1	-1.4	0.8	0.2	0.0	-0.6
R	Sep-23	-1.1	0.0	-1.3	0.9	0.5	0.0	0.9
	Sep-24	1.3	-0.2	0.0	-1.3	-1.3	0.0	-1.0
	Jul-23	-0.7	-1.1	0.9	-1.4	-1.3	-1.2	1.4
Control	Sep-23	-1.3	-1.2	1.1	-1.4	0.9	-1.2	-1.4
	Sep-24	-1.1	-1.1	0.0	0.2	0.1	-1.2	-0.3
	Jul-23	-0.7	1.3	0.5	0.6	1.1	1.2	-0.8
D	Sep-23	-1.5	1.2	0.2	0.5	-1.4	1.2	0.4
	Sep-24	-0.3	1.3	0.0	1.1	1.2	1.2	1.4
